# Plant Identity Shaped Rhizospheric Microbial Communities More Strongly Than Bacterial Bioaugmentation in Petroleum Hydrocarbon-Polluted Sediments

**DOI:** 10.3389/fmicb.2019.02144

**Published:** 2019-09-13

**Authors:** Dimitri J. Dagher, Ivan E. de la Providencia, Frédéric E. Pitre, Marc St-Arnaud, Mohamed Hijri

**Affiliations:** ^1^Institut de Recherche en Biologie Végétale, Université de Montréal and Jardin Botanique de Montréal, Montreal, QC, Canada; ^2^Neomed-Labs, Laval, QC, Canada

**Keywords:** plant-microbes interactions, microbial ecology, rhizosphere microbiome, bioaugmentation, petroleum hydrocarbon contamination, amplicon sequencing

## Abstract

Manipulating the plant-root microbiota has the potential to reduce plant stress and promote their growth and production in harsh conditions. Community composition and activity of plant-roots microbiota can be either beneficial or deleterious to plant health. Shifting this equilibrium could then strongly affect plant productivity in anthropized areas. In this study, we tested whether repeated bioaugmentation with Proteobacteria influenced plant productivity and the microbial communities associated with the rhizosphere of four plant species growing in sediments contaminated with petroleum hydrocarbons (PHCs). A mesocosm experiment was performed in randomized block design with two factors: (1) presence or absence of four plants species collected from a sedimentation basin of a former petrochemical plant, and (2) bioaugmentation or not with a bacterial consortium composed of ten isolates of *Proteobacteria*. Plants were grown in a greenhouse over 4 months. MiSeq amplicon sequencing, targeting the bacterial 16S rRNA gene and the fungal ITS, was used to assess microbial community structures of sediments from planted or unplanted microcosms. Our results showed that while bioaugmentation caused a significant shift in microbial communities, presence of plant and their species identity had a stronger influence on the structure of the microbiome in PHCs contaminated sediments. The outcome of this study provides knowledge on the diversity and behavior of rhizosphere microbes associated with indigenous plants following repeated bioaugmentation, underlining the importance of plant selection in order to facilitate their efficient management, in order to accelerate processes of land reclamation.

## Introduction

The rhizosphere microbiome usually refers to bacterial, archaeal and fungal communities as well as their genetic material closely surrounding plant-root systems (Mendes et al., 2013). The metagenome of this microbiome has been referred to as the second genome of the plant (Berendsen et al., 2012). It is a dynamic community of microorganisms, from which a part of the species have developed long-lasting intimate and specific interactions with plants roots (Vandenkoornhuyse et al., 2015). Recent advances in plant-microbe interactions demonstrated their profound effects on the growth, nutrition, and health of plants (Barea et al., 2002; Mendes et al., 2011; Hrynkiewicz and Baum, 2012; Bakker et al., 2013). The rhizosphere microbiota is complex with a dynamic spatiotemporal structure that adapts rapidly depending on biotic and abiotic stresses (Marschner et al., 2002; Mendes et al., 2013).

Recent advances in next-generation sequencing and bioinformatics allow the unraveling of taxonomic composition and functions of complex communities in a wide range of habitats and environmental conditions (Hiraoka et al., 2016). Much of our current knowledge regarding interactions and processes in the rhizosphere microbiome has emerged from studies in natural and agricultural environments (Quiza et al., 2015; Busby et al., 2017). On the other hand, the knowledge of rhizospheric microbial communities associated with plants growing under stressful conditions in highly anthropized areas is still in its early stages. Although, in this decade, there has been an increase in research attention to these habitats (Bell et al., 2014, 2016; Yergeau et al., 2014, 2015, 2018; Gonzalez et al., 2018), more studies are needed to understand how plants recruit key microbial taxa to better cope with stressful conditions, and to help design new green strategies for ecosystem restoration. For instance, land reclamation based on phytoremediation is a technique that relies on the use of plants (e.g., *Salix* spp. or hyperaccumulator plants) and their associated microorganisms to stabilize or reduce pollutants in soils (Bell et al., 2014). Although, this practice has been shown to be effective to rehabilitate mildly contaminated soils, phytoremediation efficiency might be compromised at higher levels of pollution due to poor growth of introduced plants (Pulford and Watson, 2003), caused at least in part by variations in their strength of association with resident microbes (Bell et al., 2014). An ecologically friendly and low carbon footprint method for the remediation of petroleum hydrocarbons (PHCs) is phytoremediation using a monoculture of fast growing, contaminant-tolerant high-biomass plants, with very developed root system (i.e., willows or poplars) (Pilon-Smits, 2005; Hassan et al., 2014) that can recruit and maintain an increased biological activity, which in turn will accelerate the biodegradation of the pollutants. Native plant species have been shown to develop more cooperative interactions with indigenous microbes than foreign-introduced plant species (Johnson, 2010), making the use of naturally tolerant local plants attractive for the phytoremediation of petroleum compounds. A previous survey of natural revegetation patterns in sediments highly polluted with petroleum by-products reported a high diversity of spontaneously arising plants (Desjardins et al., 2014), suggesting that many species are adapted to survive under stressful conditions. Precise information regarding their associated microbes is of paramount importance to decipher microbiome-mediated mechanisms of plant adaptation to stress, that should provide ecological services adapted to site specificity. Bioaugmentation, which is the inoculation with indigenous or allochthonous microorganisms to enhance desired biological functions, has been used in many applications, such as wastewater treatment, food production or bioremediation of polluted soil (Vogel, 1996; Limbergen et al., 1998; Singer et al., 2005). The inoculation of a given environment can affect the established indigenous microbiota by triggering and/or accelerating community shift dynamics (Morgante et al., 2010; Zhang et al., 2014).

The aim of this study was to test whether repeated bioaugmentation with bacterial consortium composed of ten isolates of *Proteobacteria*, influenced the rhizosphere microbial communities associated with the roots of four plant species growing in sediments contaminated with PHCs, as well as their productivity. To address this, we performed an experiment in a greenhouse during a four-month growth period, using mesocosms filled with petroleum hydrocarbon-polluted sediments collected from a decantation basin of a former petrochemical plant. The microcosms were planted or not with a mixture of four plant species, and were inoculated or not with a bacterial consortium of ten strains belonging to six genera of Proteobacteria that were isolated from the polluted sediments. The four plant species (*Persicaria lapathifolia*, *Lythrum salicaria*, *Lycopus europaeus*, and *Panicum capillare*) were selected among the spontaneously growing vegetation thriving in the highly polluted sedimentation basin. We hypothesized that bioaugmentation of the rhizosphere of plants already able to tolerate pollutant stress with Proteobacteria retrieved from the polluted sediments will result in an increased biodegradation of the PHCs and cause a shift in the structure of the rhizosphere microbiome toward the bioaugmented taxa.

## Materials and Methods

### Contaminated Sediments

Sediments contaminated with petroleum-hydrocarbons were collected in October 2013 from a by-product sedimentation basin of a petrochemical plant located at Varennes, on the south shore of the St-Lawrence River near Montreal, Canada (45°41′56′′N, 73°25′43′′W). Basic chemical characteristics of the sediments have been previously described (Desjardins et al., 2014). Contaminated sediments were collected from the 0–10 cm layer of the decantation basin and brought back to the laboratory where they were thoroughly homogenized and transferred into 60 × 29 × 12 cm trays to a final volume of 18 L per tray, and used as the growth substrate for plants. One kilogram of the homogenized contaminated sediments was kept at −80°C and subsequently used for bacterial isolation, as described below. At the end of the experiment and after plant harvesting, the substrate was homogenized in the trays and a composite sample made up of three subsamples was taken per each tray and conserved at −20°C until used for PHC levels measures. Initial hydrocarbons concentrations were 3055 ± 188 mg/kg for C10-C50 and 35.4 ± 2.6 mg/kg for PAHs. From here on, the contaminated sediments will be referred to as the *substrate*.

### Seeds Harvesting and Germination

Seedpods from four plant species, *P. lapathifolia*, *L. salicaria*, *L. europaeus*, and *P. capillare*, naturally growing within the same contaminated basin used to collect the sediments, were harvested in October 2013. These species were chosen based on the availability of seedpods, as well as on their germination success. The seeds were stratified in sterilized damp sand at 4°C for 8 weeks, after which they were germinated in a 1:1 (v:v) sterile sand/calcined montmorillonite clay (Turface^®^; Profile Products LLC, Buffalo Grove, IL, United States) mix incubated at room temperature (∼22°C). Germinated seeds were hand-selected and planted in 50 ml multi-cell compartments filled with an all-purpose commercial potting soil mix (Scotts Canada Ltd., Mississauga, ON, Canada) autoclaved twice (121°C for 45 min). After 3 weeks of growth, seedlings were carefully transferred to the trays containing the substrate following the design described below.

### Isolation and Identification of Bacteria From the Substrate, and Preparation of the Consortium

A 10% strength tryptic soy agar medium (3g/L) (Sigma-Aldrich, Oakville, ON, Canada) was prepared and autoclaved for 30 min at 121°C. The medium was supplemented with 100 mg/L cycloheximide before solidification to inhibit fungal growth and it was poured in Petri dishes. An inoculum was prepared by serially diluting down to 10^–7^ a thoroughly vortexed stock suspension composed of 1 g of the substrate in 9 ml of sterile demineralized water. Aliquots of 100 μl from dilutions of 10^–6^ and 10^–7^ were spread on the culture medium and incubated at 27°C for 1 week while being checked daily for bacterial growth. All growing colonies were subcultured on the same medium in order to obtain a pure culture. The bacterial isolates were then stored at 4°C on the same solid growth medium for 3 days until use. Bacterial isolates were identified by sequencing the 16S rRNA gene, which was PCR-amplified using primers 27f and 1492r (Klindworth et al., 2013). A DNA sample was picked from each colony using a 1-μl sterile inoculation loop and directly added to the PCR master mix, that was made up of 1× PCR buffer, 0.5 mg BSA, 2 mM MgCL_2_, 0.2 μM of each primer, 0.2 mM of deoxynucleotide triphosphate (dNTPs), and one unit of the Qiagen *Taq* DNA Polymerase (Qiagen, Canada) in a total volume of 20 μl per reaction. Thermal cycling conditions were as follow: initial denaturation at 94°C for 3 min; 30 cycles at 95°C for 30 s., 55°C for 30 s., and 72°C for 1 min, and a final elongation step at 72°C for 10 min. PCR reactions were performed on an Eppendorf Mastercycler ProS thermocycler (Eppendorf, Mississauga, ON, Canada). Sanger DNA sequencing was achieved using a commercial service provided by Genome Quebec Innovation Center at McGill University (Montreal, QC, Canada).

To prepare the consortium, ten bacterial isolates from six genera belonging to the phylum *Proteobacteria* were selected (Supplementary Table S1). *Proteobacteria* were chosen because they have been found as a dominant and active bacterial group within the same sediments in previous studies (Bell et al., 2014; Pagé et al., 2015; Stefani et al., 2015). Bacterial isolates were selected based on their growth vigor and stability after subculturing. Isolates were individually cultured in 2 L flasks containing each 1 L of 10% strength tryptic soy broth (3 g/L) (Sigma-Aldrich, Oakville, ON, Canada) at 27°C for 72 h with agitation, after which the liquid cultures were centrifuged at 5000 *g* for 10 min at 4°C. The resulting bacterial cell pellets were re-suspended in 1 L of a sterile isotonic 0.154 M NaCl solution. Cell counts for each isolate were performed using a Neubauer improved hemocytometer (Sigma-Aldrich, Oakville, ON, Canada), and the inoculum was made up by suspending all the selected isolates in equal amounts in a final volume of 2.4 L at a final concentration of 2.4 × 10^9^ CFU/ml.

### Experimental Design of the Mesocosm Experiment

The experiment was setup in a randomized block design in four blocks, with two factors: microcosms were planted or not, and inoculated or not, resulting in four treatment-combinations. The treatments were the following: not planted and non-inoculated (P−B−), planted and non-inoculated (P+B−), not planted and inoculated (P−B+), and planted and inoculated (P+B+). Each planted tray contained three rows, separated by 7.5 cm from each other. Within each row, the four plant species were randomly distributed in four planting positions, each being a cluster of four individual seedlings of the same plant species (Supplementary Figure S1). These clusters were placed at 12.5 cm intervals on each row. Planted and non-planted mesocosms were watered as needed, several times weekly throughout the experiment. No fertilization was applied. Bioaugmentation with the bacterial consortium was performed twice in both P−B+ and P+B+ treatments, 2 and 4 weeks after the seedlings were transplanted in the substrate. At both bioaugmentations, each tray received 300 ml of the bacterial consortium, which gave a final concentration of 4 × 10^7^ CFU/ml of dry soil, or 300 ml of sterile water as a control.

### Data Collection and Harvest

The experiment was harvested after 16 weeks of growth. At harvest, each plant cluster was carefully removed from the substrate to avoid root damage and aerial parts were separated from the roots. The substrate was gently shaken-off from the roots and the rhizospheric substrate still attached was brushed into plastic bags, flash frozen in liquid nitrogen, and kept at −80°C until DNA extraction. Soil from each plant cluster was conserved individually and represented one sample. In non-planted mesocosms, two composite soil samples were collected, each made out of three soil subsamples taken following the scheme presented in Supplementary Figure S2 and preserved similarly. Aerial plant parts were oven-dried for 72 h at 60°C before being weighted.

### DNA Extraction, PCR Amplification and Illumina MiSeq Sequencing

Total genomic DNA was extracted from 96 samples of rhizospheric substrate from the planted mesocosms (4 blocks × 2 planted treatment (P+B− and P+B+) × 12 plant clusters per tray that formed 1 sample each, and from 16 bulk substrate samples from the unplanted mesocosms (4 blocks × 2 unplanted treatments (P−B− and P−B+) × 2 composite samples per tray), using the Nucleospin^®^ Soil Kit (Macherey-Nagel Inc., Bethlehem, PA, United States), following the manufacturer’s instructions. The extracted DNA was then diluted ten folds in sterile PCR grade ultrapure water, to reduce the risk of PCR inhibition by PHCs and humic substances. A two-step PCR procedure was performed to generate amplicons of bacterial 16S rRNA and fungal ITS genes suitable for MiSeq sequencing. In the first PCR rounds, primers Bakt_341F and Bakt_805R targeting the V3-V4 region of bacterial 16S rRNA genes (Klindworth et al., 2013), and primers ITS1F and 58A2R targeting the fungal ITS-1 region (Martin and Rygiewicz, 2005) were used. To each primer was added the Illumina overhang forward and reverse adapters to which unique sample-specific indexes have been attached in the second PCR round. Amplicons were then purified using the NucleoMag^®^ NGS Clean-up and Size Select kit (Macherey-Nagel, Canada). In the second PCR round, the Nextera XT V2 Illumina MiSeq specific index kit was used to attach individual indexes to amplicons from each sample, using a limited cycles run as recommended by the manufacturer. The tagged sequences were then purified and normalized using the SequalPrep^TM^ Normalization Plate Kit, after which they were pooled at equimolar concentration and sequenced on an Illumina MiSeq sequencer using the 600 cycle MiSeq Reagent Kit v.3 in 2 × 300 bp configuration (Illumina Inc., San Diego, CA, United States). The sequences for all primers can be found in Supplementary Table S2, and the specific PCR conditions in Supplementary Section II.

### Sequence Processing and Statistical Analysis

The assembling of reads and primers trimming were done in Mothur (v.1.34.4), while the rest of the initial processing was performed in QIIME (v.1.9), following the Brazilian Microbiome Project 16S and ITS profiling pipeline (Pylro et al., 2013, 2014). Additional details of the main steps of this pipeline can be found in the Supplementary Section I. The MiSeq sequences were deposited in the NCBI Sequence Read Archive and can be found under project number PRJNA507467. The effect of bioaugmentation with the bacterial consortium on plant shoot biomass, and the effects of bioaugmentation and of the presence of plants on the concentration of C10-C50 and polycyclic aromatic hydrocarbons (PAHs) at the end of the experiment, as well as on the alpha diversity indices from the samples [Chao1 estimator (Chao, 1984), Shannon’s diversity (Magurran, 2013), and Pielou’s evenness (Pielou, 1966)], were analyzed using ANOVA or Student’s *T*-test in JMP statistical software (SAS Institute Inc.). The effects of bioaugmentation with the bacterial consortium and of the presence of plants on microbial communities in the rhizosphere were analyzed in R (v3.2.0, The R Foundation for Statistical Computing). PERMANOVA was used to test the effect of treatments on the beta diversity of microbial communities in the substrate, followed by a Permutational multivariate analyses of dispersion (PERMDISP) of the Bray–Curtis matrices of these communities to assess whether any significant difference detected was effectively a shift in the communities or just due to random dispersion of the samples (Supplementary Table S3). Redundancy analysis (RDA) was performed using the “rda” function from the “vegan” v2.5 package in R, where OTU composition and relative abundance were constrained by plant presence, plant species and bioaugmentation. In order to assess which microbial taxa’s relative abundance is significantly modified by the treatments, we used the Kruskal–Wallis non-parametric analysis followed by a false discovery rate (FDR) adjustment of the *P*-value using the Benjamini–Hochberg procedure. The new *P*-value threshold (*q*-value) is 0.05 for both fungi and bacteria. Information regarding the settings used in JMP and the R code are shown in Supplementary Section III.

## Results

### Microbial Diversity in the Substrate

Sequencing of 16S rRNA gene amplicons generated a total of 1913451 reads, of which 291000 sequences with an average length of 399 ± 69 bp were retained after quality filtering and subsampling to 3000 sequences per sample. Fifteen samples did not meet the 3000 sequences cutoff and were ignored for the further analysis. Reads per sample initially ranged from 1309 to 39984 reads, and grouped after subsampling into 5876 operational taxonomic units (OTUs) at the 97% similarity threshold. The data matrix is shown in Supplementary Table S4. Good’s coverage indices ranged between 81.8 and 84.6% for all treatments after subsampling, indicating that most bacterial diversity in each sample was captured (Supplementary Figure S3).

Sequencing of ITS1 fragment yielded 1407783 sequences with an average length of 324 ± 28 bp, of which 424000 sequences were retained following quality filtering and subsampling to 4000 sequences per sample. Six samples did not meet the 4000 sequence threshold and were excluded from the analysis. Read counts ranged between 945 and 31335 per sample, and grouped after subsampling into 889 OTUs at a cut-off threshold of 97%. Goods coverage index values ranged between 98.8 and 99.2% across all treatments after subsampling, showing that nearly all the fungal diversity in each sample was captured (Supplementary Figure S4).

Bacterial Chao1 species richness in the substrate was significantly influenced by the presence of plants (P+B−, P+B+; *P* > —t— = 0.0123), compared with the unplanted treatments (P−B−, P−B+), while bioaugmentation did not affect Chao1 species richness (Supplementary Figure S5). Plants also significantly increased the Shannon diversity index (*P* > —t— < 0.0001, Supplementary Figure S6) and evenness (*P* > —t— < 0.0001, Supplementary Figure S7) of bacterial communities.

**FIGURE 1:**
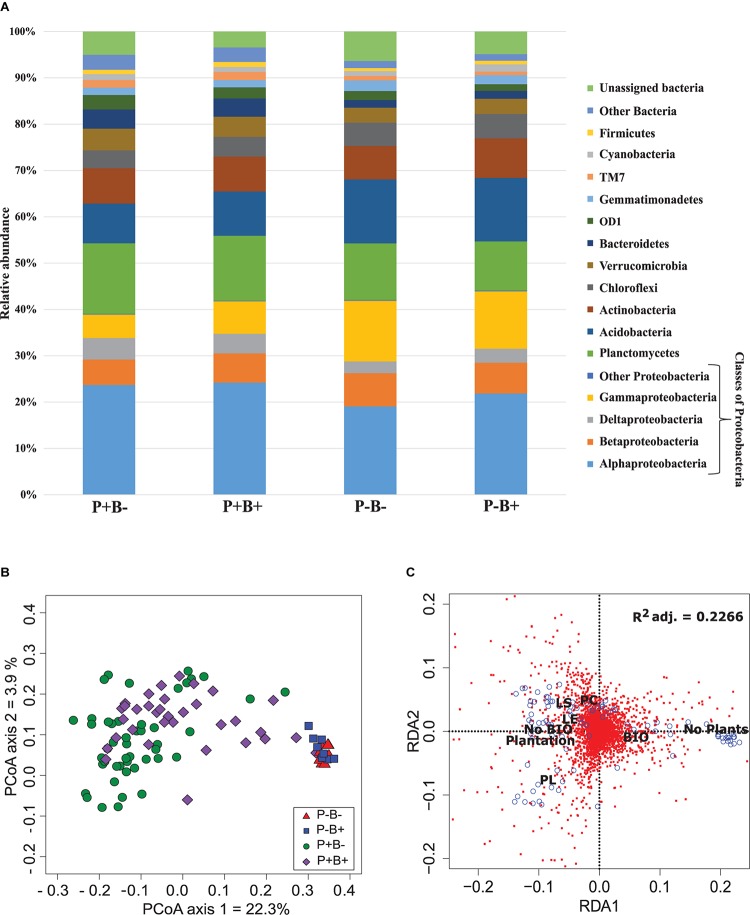
**(A)** Effect of the plantation and bioaugmentation on the relative abundance of bacterial phyla or classes. **(B)** Principal coordinate analysis based on the Bray–Curtis dissimilarity of bacterial communities showing the effect of plantation and bioaugmentation. **(C)** Redundancy analysis (RDA) showing the relation between the abundance of bacterial OTUs and the factors Bioaugmentation, Plantation, and Plant species identity. The adjusted *R*^2^ value indicates the amount of variance in bacterial community composition accounted for by the constraining factors (Bioaugmentation, Plantation, and Plant species identity). The location of labels represent factor centroids. Red squares indicate bacterial OTUs. Blue open circles represent individual samples. (Bio, Bioaugmentation; LE, *Lycopus europaeus*; LS, *Lythrum salicaria*; PC, *Panicum capillare*; PL, *Persicaria lapathifolia*).

Overall, *Alphaproteobacteria* were the most abundant group across all treatments with an average of 22% of the bacterial community, followed by *Planctomycetes* at 13% and *Acidobacteria* with 11.4%. Then, *Gammaproteobacteria* formed 9.4% of the community, *Actinobacteria* 7.7%, *Betaproteobacteria* 6.4%, *Chloroflexi* 4.6%, *Verrumicrobia* 3.9%, and *Deltaproteobacteria* 3.6% (Figure 1A). *Deltaproteobacteria*, *Planctomyces*, and *Bacteroidetes*, as well as *Alphaproteobacteria*, *Acidobacteria*, *Betaproteobacteria*, and *Gammaproteobacteria* had significantly different relative abundances in both planted treatments than in the unplanted mesocosms.

For fungi, Chao1 richness estimator was significantly lower in planted than unplanted mesocosms (*P* > —t— < 0.0001), indicating that plant rhizosphere contained a lower number of fungal taxa than unplanted substrate (Supplementary Figure S8). On the other hand, Pielou’s evenness was significantly increased in the presence of plants (*P* > —t— = 0.003; Supplementary Figure S9). The Shannon diversity index did not change significantly in any of the treatments (Supplementary Figure S10).

**FIGURE 2:**
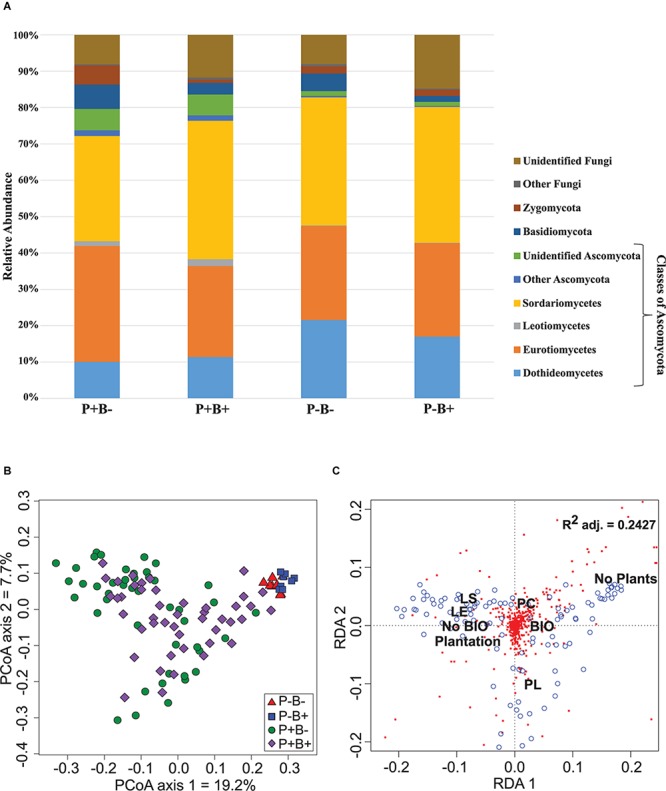
**(A)** Effect of the plantation and bioaugmentation on the relative abundance of fungal phyla or classes. **(B)** Principal coordinate analysis based on the Bray–Curtis dissimilarity of fungal communities showing the effect of plantation and bioaugmentation. **(C)** Redundancy analysis (RDA) showing the relation between the abundance of fungal OTUs and the factors Bioaugmentation, Plantation, and Plant species identity. The adjusted *R*^2^ value indicates the amount of variance in fungal community composition accounted for by the constraining factors (Bioaugmentation, Plantation, and Plant species identity). The location of labels represents factor centroids (Bio, Bioaugmentation; LE, *Lycopus europaeus*; LS, *Lythrum salicaria*; PC, *Panicum capillare*; PL, *Persicaria lapathifolia*). Red squares indicate bacterial OTUs. Blue open circles represent individual samples.

*Ascomycota* dominated the substrate in all treatments, and formed 79.6% to 84.5% of the fungal community (Figure 2A). *Basidiomycota* ranged between 1.5 and 6.7%, and they showed significantly relative abundances in the bioaugmented mesocosms, regardless of the presence of plants (FRD-adj. *P* = 0.04). *Sordariomycetes* abundances were also significantly different in the bioaugmented mesocosms, but only in the presence of plants (P+B+; FRD-adj. *P* = 0.03), and formed between 28 and 38% of the community. Unknown fungi formed between 14.8 and 8% of the community and the rest of the fungal diversity not mentioned here ranged from 0.4% to 1.5%.

### Effect of Bioaugmentation and Plantation on Microbial Community Structures

A clear influence of the presence of plants in the bacterial communities’ assemblages was revealed by principal coordinate analysis (PCoA) based on Bray–Curtis dissimilarity indices, with samples from unplanted and planted microcosms mostly clustering on opposite sides of the biplot (Figure 1B). PERMANOVA analysis based on the Bray–Curtis dissimilarity matrix of bacterial communities showed that inoculation, presence of plants, and plant species identity all significantly influenced the rhizospheric bacterial community structure (Table 1). The presence of plants explained 14.1% of the observed variation in community structure, followed by plant species identity (Supplementary Figure S11) and bioaugmentation with 5.5 and 3.3%, respectively, as accounted by the *R*^2^ values (Table 1). PERMDISP were conducted to test for differences in bacterial community dispersion between treatments, since a significant PERMANOVA result may indicate either a difference in centroids or an unequal dispersion between treatments. Results showed that the dispersion in bacterial communities was not significantly affected among planted treatments (Supplementary Figure S12), supporting that the difference detected by PERMANOVA was due to a shift in community composition. RDA analysis of the bacterial communities confirmed the stronger influence of plantation on the abundance of bacterial OTUs, and also showed that plant species identity had a differential influence on it (Figure 1C).

**TABLE 1:** PERMANOVA analysis of the effects of the plantation, plant species identity and bioaugmentation on bacterial community structure in the substrate based on the Bray–Curtis dissimilarity indices.

**Factor**	**Df**	**SumsOfSqs**	**MeanSqs**	***F*.Model**	***R*^2^**	**Pr( > *F*)**
Bioaugmentation	1	0.5015	0.50154	3.9262	0.03276	0.001^∗^
Plantation	1	2.1622	2.16222	16.9264	0.14122	0.001^∗^
Plant species	3	0.8382	0.27939	2.1871	0.05474	0.002^∗^
Bioaugmentation: plantation	1	0.1747	0.17475	1.368	0.01141	0.094
Bioaugmentation: plant Species	3	0.5203	0.17344	1.3578	0.03398	0.051
Residuals	87	11.1136	0.12774		0.72588	
Total	96	15.3106			1	

*Df, degree of freedom; SumsOfSqs, sums of squares; Meansqs, mean squares; *F*.Model, *F*-test value for model; *R*^2^, *R*-squared; Pr(>F), *p*-value.*

In the case of fungi, the PCoA of the Bray–Curtis dissimilarity indices showed that fungal communities in planted (P+B−, P+B+) distributed on the left side of the biplot while those from unplanted treatments (P−B−, P−B+) tightly clustered together on the right side of the biplot, regardless of the bioaugmentation treatment (Figure 2B). PERMANOVA analysis showed that presence of plants, inoculation, and plant species identity all significantly influenced the fungal rhizospheric communities (Table 2), with both plant presence and plant identity (Supplementary Figure S13) explaining 10.9% of the observed variation, followed by inoculation explaining 3.6% of the variation (Table 2). There was also a significant interaction between bioaugmentation and plant species identity, which was responsible of 3.5% of the variation in community structure (Table 2). PERMDIPSD analysis showed that the dispersion of the fungal rhizospheric communities was not significantly different between the unplanted and planted mesocosms (Supplementary Figure S14), which confirms that the differences detected by the PERMANOVA were due to shifts in community structure. As with bacteria, RDA analysis confirmed that plantation had a strong relationship with the abundances of fungal OTUs, which also related differentially with plant species identity (Figure 2C).

**TABLE 2:** PERMANOVA analysis of the effects of the plantation, plant species identity and bioaugmentation on fungal community structure in the substrate, based on Bray–Curtis dissimilarity indices.

**Factor**	**Df**	**SumsOfSqs**	**MeanSqs**	***F*.Model**	***R*^2^**	**Pr(>F)**
Bioaugmentation	1	0.5675	0.56755	4.9734	0.03623	0.001^∗^
Plantation	1	1.7199	1.71989	15.0713	0.10978	0.001^∗^
Plant species	3	1.7212	0.57374	5.0276	0.10987	0.001^∗^
Bioaugmentation: plantation	1	0.1512	0.15118	1.3248	0.00965	0.102
Bioaugmentation: plant species	3	0.551	0.18366	1.6094	0.03517	0.005^∗^
Residuals	96	10.9552	0.11412		0.6993	
Total	105	15.6661			1	

*Df, degree of freedom; SumsOfSqs, sums of squares; Meansqs, mean squares; *F*.Model, *F*-test value for model; *R*^2^, *R*-squared; Pr(>F), *p*-value.*

### Effect of Treatments on Plant Shoot Biomass and Petroleum Hydrocarbon Concentrations

**FIGURE 3:**
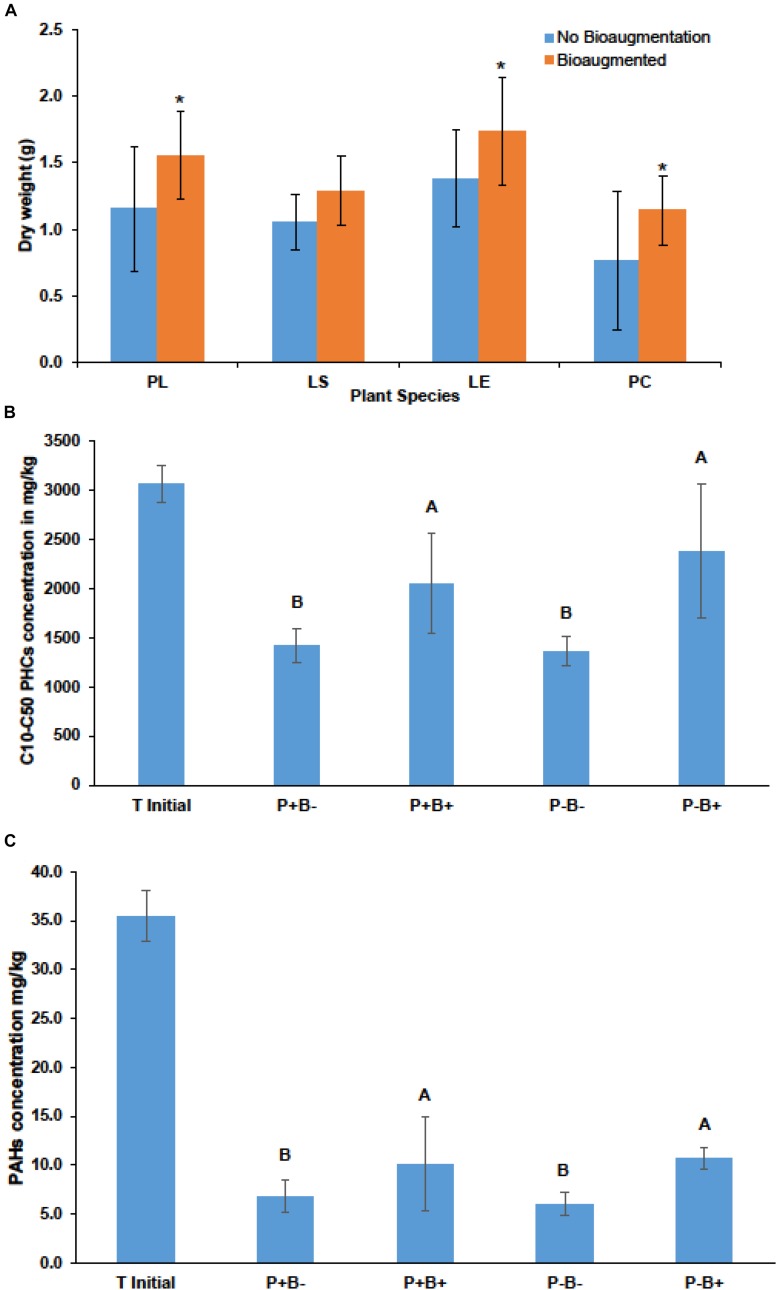
**(A)** Effect of bioaugmentation on average plant shoot dry biomass for each species at the end of the experiment. Errors bars are standard deviation. PL, *Persicaria lapathifolia*; LS, *Lythrum salicaria*; LE, *Lycopus europaeus*; PC, *Panicum capillare*. *N* = 12 for each species. Average dry weights for each species were compared with or without bioaugmentation using a Student’s *T*-test. Asterisks indicate that the dry weight was significantly different between treatments. **(B)** Average remaining concentration in the sediments of C10–C50 aliphatic hydrocarbons in each treatment. **(C)** Average remaining polycyclic aromatic hydrocarbons (PAHs) at the end of the experiment. Errors bars represent standard deviation. *N* = 4 for each treatment. For **(B,C)** plantation and bioaugmentation effects were tested using a two-way full factorial ANOVA in JMP v.7. Treatments not sharing the same letter are significantly different. We added the initial value for reference.

Bioaugmentation significantly increased the average shoot dry weight across all plant species in comparison to the non-bioaugmented controls except for *L. salicaria* (*P* = 0.0001) (Figure 3A). Bioaugmentation also influenced the concentrations of aliphatic hydrocarbons (C10–C50 fraction) and aromatic polycyclic hydrocarbons (PAHs) in the substrate, at the end of experiment (Figures 3B,C). Intriguingly, there was significantly less (*P* = 0.0088) aliphatic PHCs in the non-bioaugmented treatments compared to bioaugmentation treatments (Figure 3B). Values ranged from 1360 ± 147 mg/kg in the non-inoculated treatments to 2660 ± 686 mg/kg in the planted bioaugmented treatments. The same trend was observed for PAHs (*P* = 0.0181) (Figure 3C), with levels ranging from 6 ± 1.14 mg/kg in the non-bioaugmented treatments to 10.7 ± 1.11 mg/kg in the planted and bioaugmented substrate. Plantation did not have any significant effect on both final C10–C50 hydrocarbons and PAH concentrations.

## Discussion

In this study, we determined the effect of the bioaugmentation with a consortium of *Proteobacteria* on the productivity and rhizosphere microbial communities of four spontaneous plant species growing together in sediments contaminated with PHCs. We found that the presence of plants and plant species identity were the main drivers of bacterial and fungal communities in the substrate. Bioaugmentation also significantly shift microbial community structure and increased the overall total plant shoot dry biomass, but reduced PHC degradation even in the unplanted bulk substrate. Therefore our hypothesis regarding the bioaugmentation of the rhizosphere and increased biodegradation of the PHCs is rejected.

### Plantation Shaped the Rhizosphere Microbiome

Plants have been shown to be strong drivers of the abundance and structure of soil microbial communities. They can recruit and influence the abundance of soil microorganisms through root exudation of carbon compounds and other mechanisms (Kowalchuk et al., 2002; Berg et al., 2005; Berendsen et al., 2012; Mendes et al., 2013; Peiffer et al., 2013; Bucci, 2015), making the rhizosphere a highly selective environment. In our study, bacterial and fungal communities’ structures were significantly different between the rhizosphere and unplanted bulk substrate. Results showed that plants shaped significantly the bacterial and fungal communities under polluted conditions, thus altering the microbiome composition. Plants can attract rhizosphere colonizers in their direct vicinity by depositing a myriad of compounds and substances, however, it is still unclear which mechanisms are used by plants to modify their exudation profile to recruit microbes. Studies using DNA Stable Isotope Probing approach (DNA-SIP) (Haichar et al., 2008), whole genome transcriptome approach (Mark et al., 2005), and whole genome microarray (Shidore et al., 2012) have clearly demonstrated changes in bacterial transcriptional profiles under the influence of root exudates, which were also shown to influence soil fungal community composition and diversity (Broeckling et al., 2008), underlining the role of plants in driving rhizospheric bacteria and fungi (Hartmann et al., 2009).

Attracting microbes in their rhizosphere can benefit plants in multiple ways because microbes are responsible for the acquisition of 5–80% of nitrogen, and up to 75% of phosphorus that is necessary for plant growth and development (Heijden et al., 2008). Here, we found that among the 50 most abundant bacterial OTUs in the plant rhizospheres, nine were assigned to the order *Rhizobiales* versus none in the unplanted bulk substrate (Supplementary Table S2). Bacteria of this group are considered as plant growth-promoting rhizobacteria, that are able to enhance plant growth by increasing the nitrogen availability (Zahran, 2001). The second most abundant OTU in the plant rhizosphere was identified as *Kaistobacter* sp. The *Kaistobacter* genus was shown to be associated with the suppression of tobacco bacterial wilt caused by *Ralstonia solanacearum* (Liu et al., 2016). Plants can also benefit from associations with fungi. Strains belonging to the genera *Fusarium*, *Penicillium*, *Phoma*, and *Trichoderma* have been shown to induce systemic resistance in cucumbers, which lead to a greater resistance to multiple diseases (Koike et al., 2001; Benhamou et al., 2002). Moreover, some fungi were also reported to increase plant nutrients mobilization and uptake, as it is the case with symbiotic fungi such as the arbuscular mycorrhizal fungi, which can solubilize and translocate nutrients from the soil to the plant roots through their vast mycelial network (Parniske, 2008). Free living fungi are also able to increase nutrient availability. A strain of *Trichoderma harzianum* (Altomare et al., 1999), and other fungi such as *Trichosporon beigelii*, *Phichia norvegensis*, *Cryptococcus albidus* var *aerius*, as well as *Penicillium bilaii* (Kucey, 1987; Cunningham and Kuiack, 1992; Gizaw et al., 2017) have shown inorganic phosphate and other mineral solubilization capabilities. In our results, we observed that many of the 50 most abundant rhizospheric fungal OTUs were related to *Phoma*, *Fusarium*, *Penicillium*, *Eupenicillium*, and *Trichoderma* species (Supplementary Table S3). These results suggest that plants naturally occurring in PHC contaminated sediments recruit potential growth and health promoting microorganisms. It should be noted, however, that these genera also contain known plant pathogens.

### Plant Species-Specific Effects on Microbiome Structure

Our results also showed that plants exerted species-specific effects on the structure of rhizospheric microbial assemblages (Supplementary Figures S9, S11), highlighting the differential selectivity of the plant rhizosphere. A phenomenon known as plant-soil feedback proposes that plants, through specific root-compounds, influence soil chemical properties and soil microbial profiles which subsequently influence plant growth and productivity (Bezemer et al., 2006). Exudates from plant-roots, which include amino acids, organic acids, and carbohydrates, are differentially deposited in the rhizosphere, depending on the plant species identity and plant development stage in a given environment (Hertenberger et al., 2002; Chaparro et al., 2013). This, in turn, would lead to distinct rhizospheric microbial community structures. A terminal-restriction fragment length polymorphism analysis of the rhizospheric microbial communities of seven coastal angiosperm congeners showed that plant species significantly correlated with the variation of the rhizosphere microbiome composition (Burns et al., 2015). In another recently published study, Schmid et al. (2018) the authors grew offsprings of eight plant species that had been growing for 11 years in the field under monoculture and mixture planting. They performed 16S rRNA sequencing and found that the bacterial community structure in the rhizosphere of plants was determined by soil plantation history (monoculture vs. mixture) and plant species identity (Schmid et al., 2018). In a context of phytoremediation of PHCs, the aim is to use the plant rhizosphere as stimulator of microbial activity, which will then biodegrade the petroleum compounds (Pilon-Smits, 2005). The approach of using multiple plant species for the phytoremediation PHC compounds might not always prove to be the most effective, since different plant species might recruit different rhizospheric microbiomes, which might result in mitigated efficiency. Physico-chemical properties are the main drivers of the soil and rhizosphere microbiome (Fierer, 2017), but in a given environment, plant species can have an influence on the microbiome structure (Hartmann et al., 2009; Bell et al., 2014; Albert et al., 2015; Bulgarelli et al., 2015).

### Bioaugmentation Induced a Shift in the Rhizosphere Microbiome Structure and Increased Plant Growth

Bioaugmentation is a common approach used for the remediation of contaminated environments which resulted in a variable and contradicting conclusions (Thompson et al., 2005). These inconsistencies were attributed (1) to the performance of microbial strains used in the inoculum, as successful bioaugmentation being linked to the survival, persistence, and function of the selected organisms (Tyagi et al., 2011), (2) to the potential competition between the inoculum members and the resident community (Pala et al., 2002; Trindade et al., 2002; Mariano et al., 2009), but also to the aeration, nutrient content and soil type (Mrozik and Piotrowska-Seget, 2010).

Here, we observed that bioaugmentation caused a shift in the rhizosphere microbiome structure (Supplementary Figures S5, S6). However, decrease of hydrocarbon pollutants in non-bioaugmented microcosms (P+B−) and P−B−) was significantly higher than in bioaugmentation treatments P−B+ and P+B+ (Figures 3A,B), suggesting that the biodegradation of hydrocarbons was less effective following the repeated amendments with the bacterial consortium. The consortium candidates were selected based on previous studies on the site (Bell et al., 2014; Stefani et al., 2015) which showed that *Proteobacteria* were the dominant group in these sediments. However, isolation usually recover from 2 to 5% of the total species richness in any given site (Stefani et al., 2015). Moreover, even when their capacity as hydrocarbon degraders is demonstrated, the persistence and activity of bioaugmented strains depends fundamentally on their ability to compete with indigenous microorganisms (Thompson et al., 2005), in spite of their concentration increase through the inoculation. Similar results had already been reported previously (Yu et al., 2005; Tongarun et al., 2008; Cunningham et al., 2009) and were attributed to biotic factors like predation and competition for limited carbon sources and space through different mechanism such as antibiotic production and resistance (Hibbing et al., 2010). In our study, several of the top 50 bacterial OTUs in the bioaugmented rhizosphere were related to taxa that have been identified as plant growth promoting such as *Kaistobacter* sp., *Devosia* sp., and other *Rhizobiales* spp. (Supplementary Table S2). Similarly, plant beneficial fungi such as *Trichoderma* sp. were found among the most 50 abundant fungal OTUs in both the bioaugmented and non-bioaugmented treatments (Supplementary Table S5). The significantly higher abundance of some of these beneficial groups such as *Trichoderma* and *Kaistobacter* sp. in the bioaugmented treatments might explain the positive effects on final plant shoot biomass in comparison with the non-bioaugmented treatments (Figures 2A,B). The positive effects might be related to different mechanisms, such as the production of phytohormones, protection from environmental toxicity and pathogens, as well as increased nutrient bioavailability (Compant et al., 2005; Berg and Hallmann, 2006; Zaidi et al., 2006).

## Conclusion

Plants significantly influenced the structure of the microbial communities in the PHCs -contaminated sediments, where taxa related to plant growth promoting microorganisms where among the most abundant OTUs. Additionally, plant species identity had a significant impact on the structure of rhizosphere microbiome, highlighting the importance of plant selection in phytoremediation strategies through the creation of new niches allowing introduced organisms to persist. On the other hand, while the bioaugmentation influenced the structure of both fungal and bacterial communities in the rhizosphere, its effect was much weaker than the presence of plants and their identity which contradicts our hypothesis. One caveat is the use of a restricted set of organisms and the results cannot be generalized. There is a need for long-term *in situ* studies involving the use of autochthonous multiple-plant species compared to monoculture of species whose survival and remediation efficiency have been shown to decrease in highly contaminated environments (Pulford and Watson, 2003). However, any strategy regarding soil remediation should also consider both the degradation potential of the selected microorganisms and the interaction with host plants and the local microbiome. These studies will help to precisely select microorganisms which will improve plant health while accelerating the remediation process.

## Data Availability

The datasets generated for this study can be found in the PRJNA507467.

## Author Contributions

DD and IP designed and performed the experiment. IP, FP, MS-A, and MH supervised the project. DD analyzed the data. MS-A and MH provided the material and analytic tools. DD, IP, FP, MS-A, and MH wrote the manuscript.

## Conflict of Interest Statement

The authors declare that the research was conducted in the absence of any commercial or financial relationships that could be construed as a potential conflict of interest.
