# Supplementary material for: Plant Identity Shaped Rhizospheric Microbial Communities More Strongly Than Bacterial Bioaugmentation in Petroleum Hydrocarbon-Polluted Sediments
Source: Front Microbiol. 2019 Sep 13;10:2144. doi: 10.3389/fmicb.2019.02144 (PMC6753587; doi:10.3389/fmicb.2019.02144)
Supplement: Supplementary file 1 [file Data_Sheet_1.docx]

**Supporting information**

**Figure S1.** Experimental design and description of the plantation pattern in the mesocosms. Each tray contained twelve planting sites which consisted of a cluster of four individual plants of the same species for a total of four plant species with three planting sites each.

**Figure S2.** Sampling scheme for non-planted treatments

**
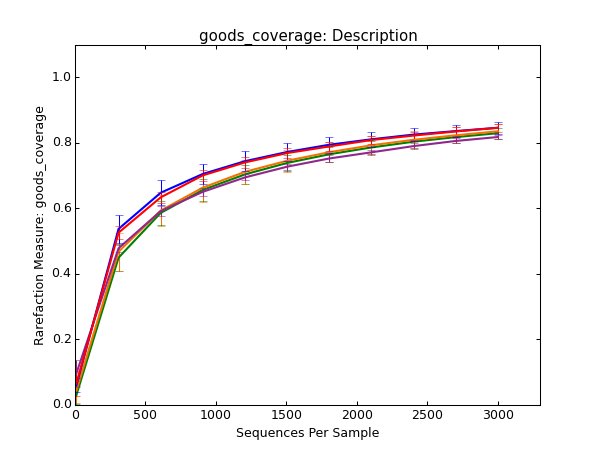
**

**Figure S3.** Good’s coverage curve of the 16S sequences after subsampling to 3000 sequences per sample.

**
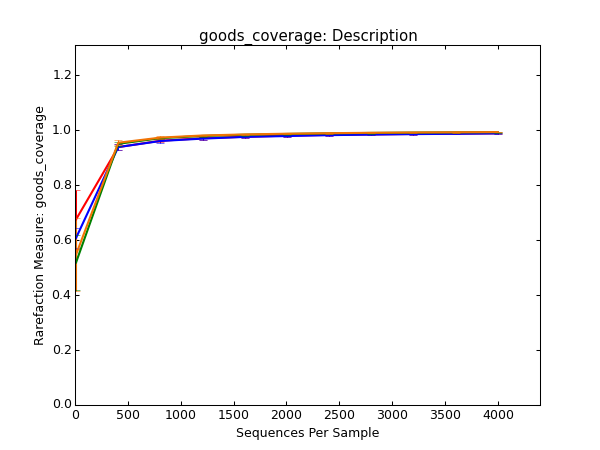
**

**Figure S4.** Goods coverage curve of the ITS sequences after subsampling to 4000 sequences per sample.

**
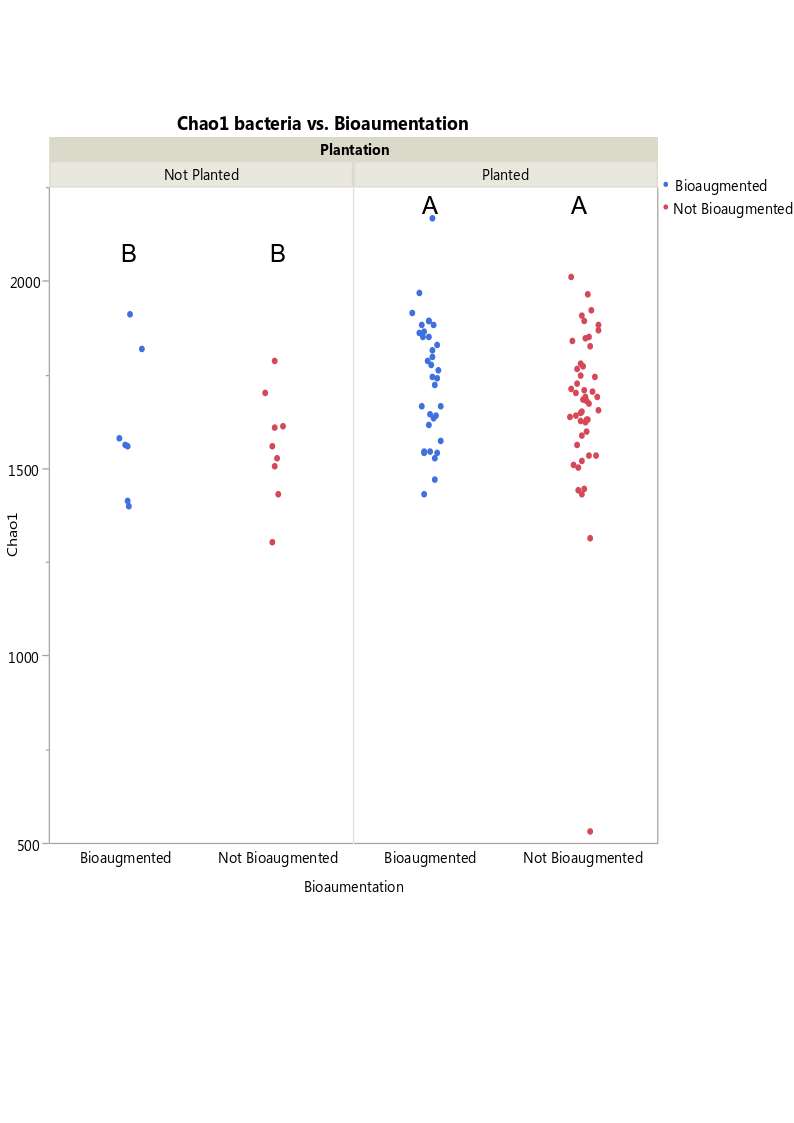
Figure S5.** Chao1 estimate for bacterial communities in all samples across all treatments. Plantation and bioaugmentation effects were tested using a two-way full factorial ANOVA in JMP v.7. Treatments not sharing the same letter are significantly different.

**
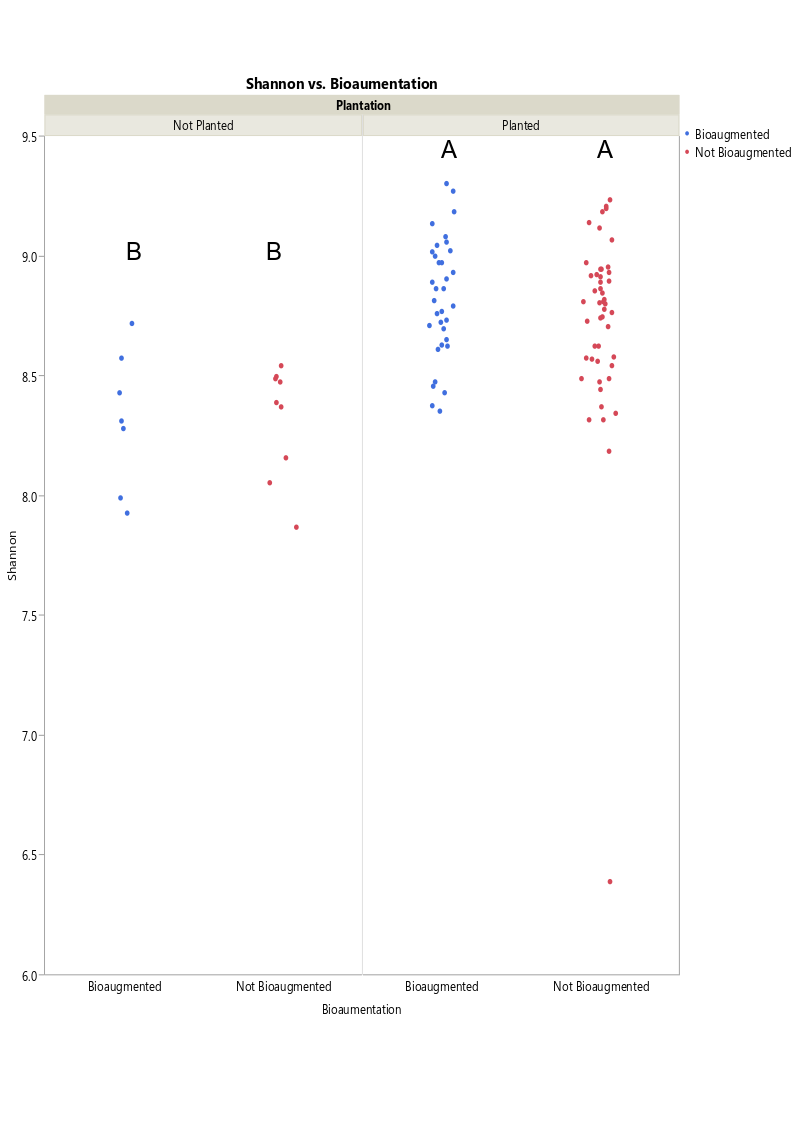
**

**Figure S6.** Shannon’s diversity index for bacterial communities in all samples across all treatments. Plantation and bioaugmentation effects were tested using a two-way full factorial ANOVA in JMP v.7. Treatments not sharing the same letter are significantly different.

**
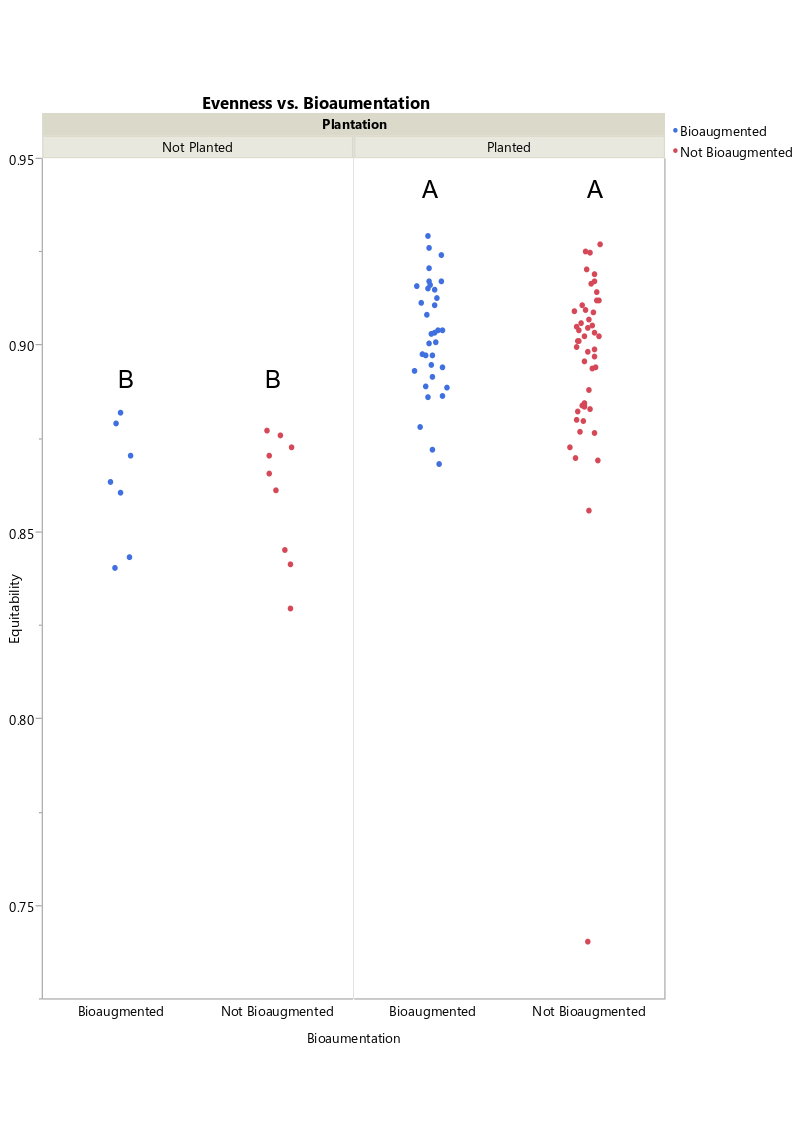
**

**Figure S7.** Pielou’s evenness measure for the bacterial communities in all samples across all treatments. Plantation and bioaugmentation effects were tested using a two-way full factorial ANOVA in JMP v.7. Treatments not sharing the same letter are significantly different.


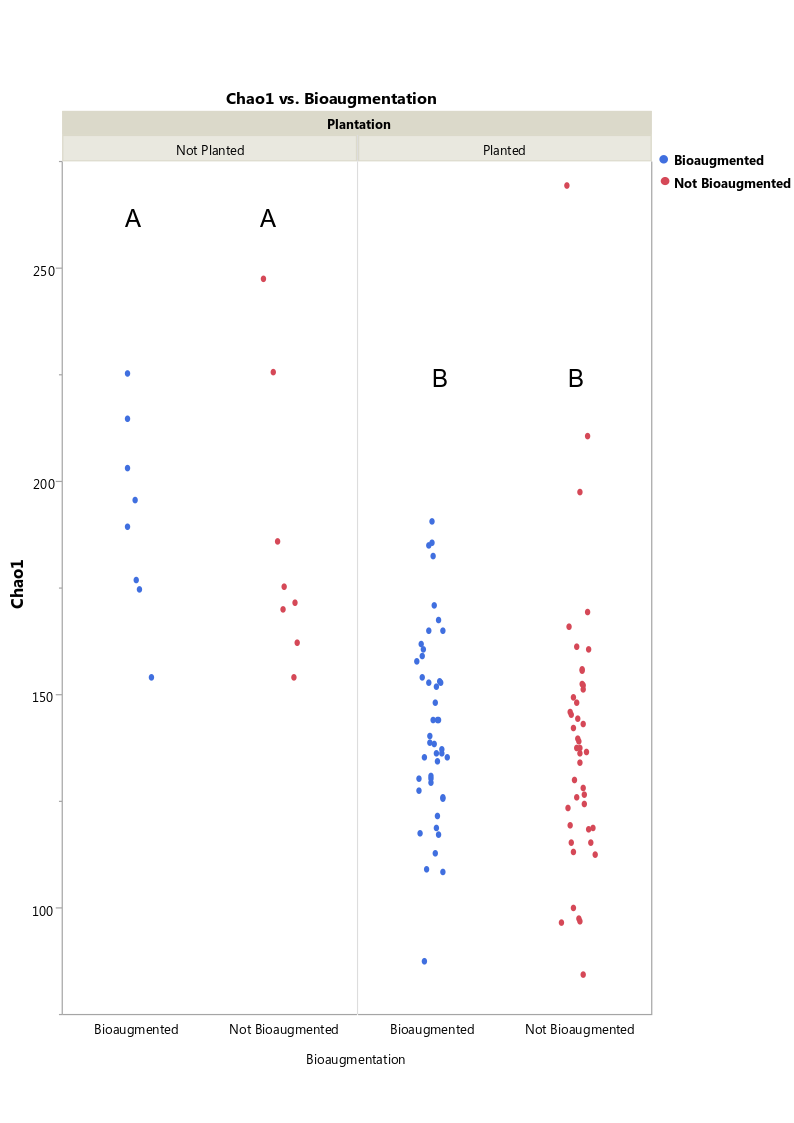


**Figure S8.** Chao1 richness estimate for fungal communities across the four treatments. Plantation and bioaugmentation effects were tested using a two-way full factorial ANOVA in JMP v.7. Treatments not sharing the same letter are significantly different.

**
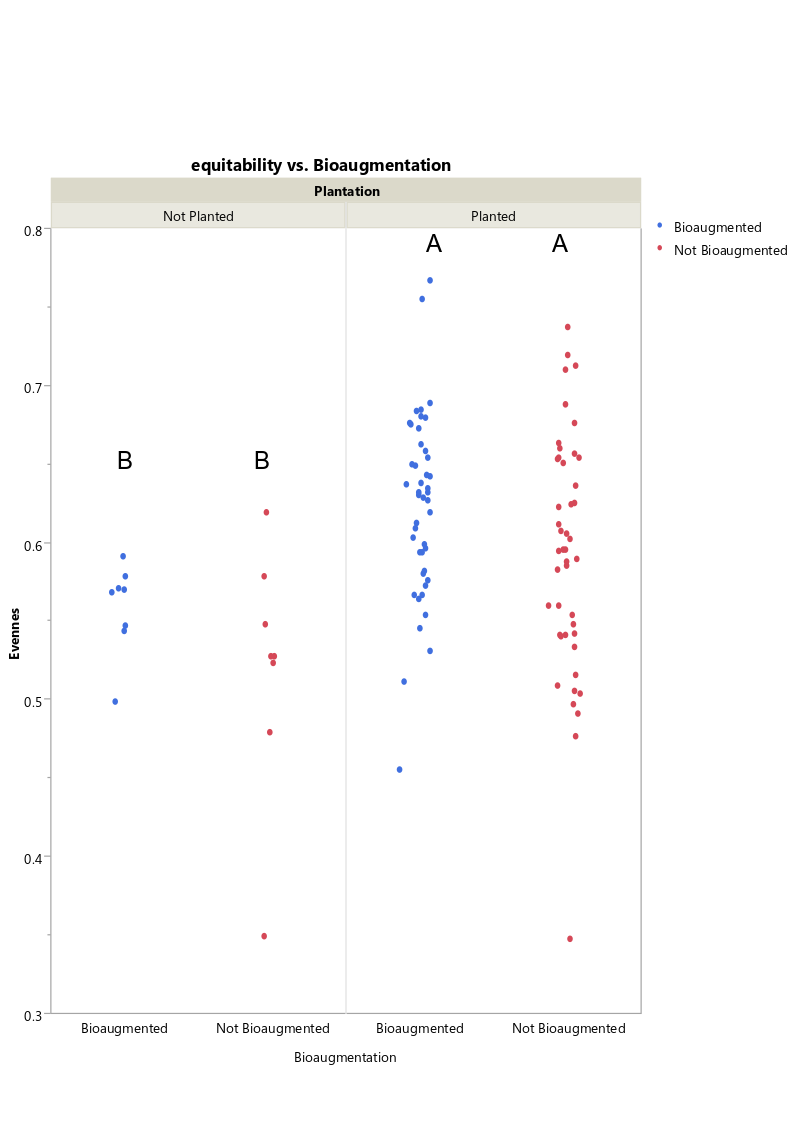
**

**Figure S9.** Pielou’s evenness measure for the fungal communities in all samples across all treatments. Plantation and bioaugmentation effects were tested using a two-way full factorial ANOVA in JMP v.7. Treatments not sharing the same letter are significantly different.

**
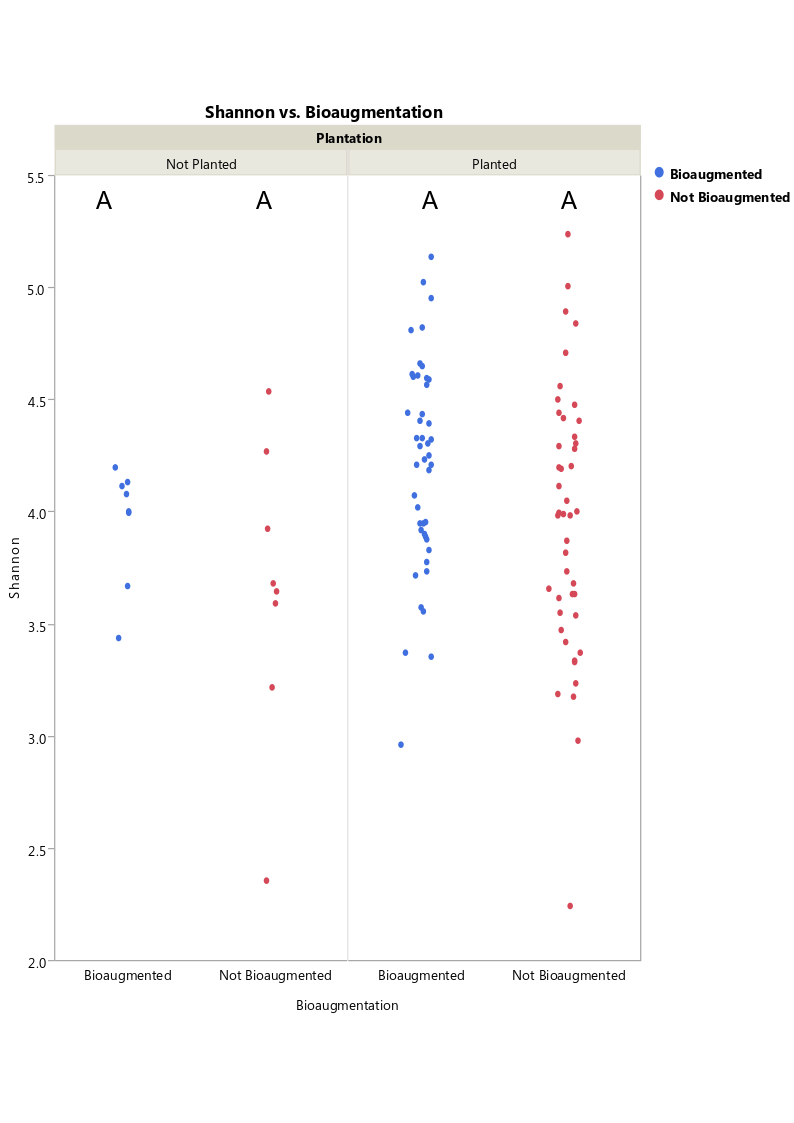
**

**Figure S10.** Shannon’s diversity index for fungal communities in all samples across all treatments. Plantation and bioaugmentation effects were tested using a two-way full factorial ANOVA in JMP v.7. Treatments not sharing the same letter are significantly different.

**
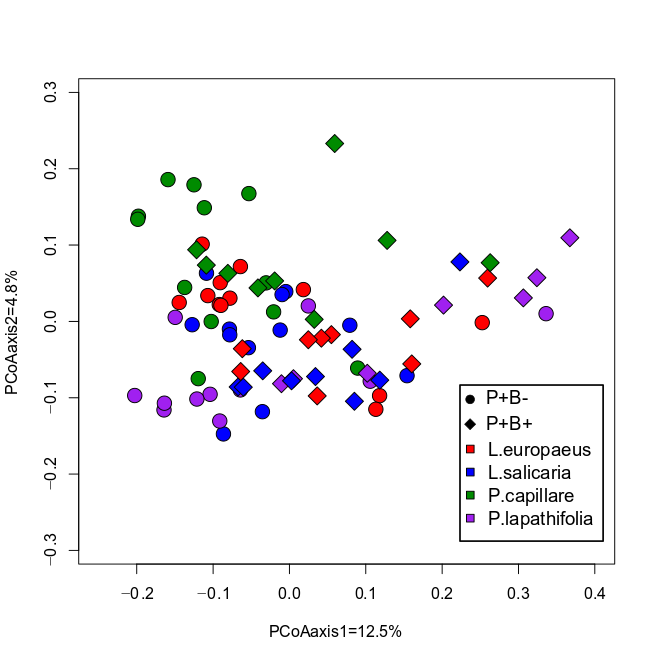
Figure S11.** Principal coordinate analysis based on the Bray-Curtis dissimilarity of rhizospheric bacteria communities. **P+B-:** Planted and not bioaugmented, **P+B+:** Planted and bioaugmented.

**Figure S12.** Boxplot of distance to centroid based on beta-dispersion analysis of bacterial community Bray–Curtis distance in both planted treatments.


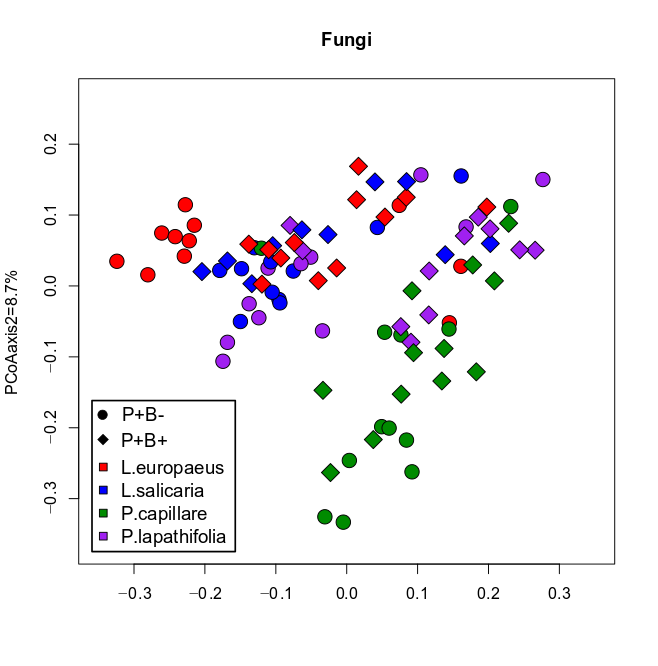
**Figure S13.** Principal coordinate analysis based on the Bray-Curtis dissimilarity of rhizospheric fungal communities. **P+B-:** Planted and not bioaugmented, **P+B+:** Planted and bioaugmented.

**Figure S14.** Boxplot of distance to centroid based on beta-dispersion analysis of fungal community Bray–Curtis distance in both planted treatments.

**Section I**

**Details of the sequence processing pipeline**

**I.A: 16S rRNA sequences pipeline**

In Mothur, the reads from each sample were assembled using the ‘make.contigs’ command. This generated a .fasta file containing the assembled reads. Primers were then removed with ‘trim.seqs’, after which he sequences were exported to QIIME. Special labels were then added for compatibility, with the ‘add.qiime.labels.py’ command. Using the Usearch7 sequence analysis tool QIIME implementation, the dataset was reduced to unique sequences using ‘–derep_fulllength’, which we sorted by decreasing cluster size, and removed singletons with ‘-sortbysize’. The sequences were then clustered by OTU (Operational Taxonomic Unit) using a 97% identity threshold using the UPARSE method, and aligned using QIIME’s implementation of the GreenGenes bacterial database 08/13 update. Further sequencing errors were removed with ‘–uchime_ref’. OTU’s were assigned taxonomic identity using the UCLUST method and GreenGenes database with ‘assign_taxonomy.py’. We then produced an OTU table at 97% similarity and sequences that classified as ‘Mitochondria’, ‘Chloroplast’, ‘Archaea’, ‘Eukaryota’, or ‘unknown’ were removed with ‘filter_taxa_from_otu_table.py’. Finally, we subsampled the sequences, so each sample had the same amount (3000). Basic alpha diversity indices were generated using the “core_diversity_analyses.py” command. The OTU table was then exported to R for diversity and statistical analyses.

**I.B: Fungal ITS sequences processing pipeline**

In Mothur, the reads 1 and 2 from each sample were assembled using the ‘make.contigs’ command. This generated a .fasta file containing the assembled reads. Primers were then removed with ‘trim.seqs’, after which the sequences were exported to QIIME. Special labels were then added for compatibility, with the ‘add.qiime.labels.py’ command. Using the Usearch7 sequence analysis tool QIIME implementation, the dataset was reduced to unique sequences using ‘–derep_fulllength’, which we sorted by decreasing cluster size, and removed singletons with ‘-sortbysize’. ITS1 sequences were then selected with the ITSx Tool, and clustered by OTU (Operational Taxonomic Unit) using a 97% identity threshold with UPARSE method. Further sequencing errors were removed with ‘–uchime_ref’. OTU’s were assigned taxonomic identity using BLAST method on QIIME and the UNITE reference database with ‘assign_taxonomy.py’. We then produced an OTU table at 97% similarity and only sequences that classified as ‘Fungi’ were retained. Basic alpha diversity indices were generated using the “core_diversity_analyses.py” command. The OTU table was then exported to R for diversity and statistical analyses.

**Section II**

**PCR conditions**

**16S amplification**

**First round reagents**

| **Component** | **Final concentration** |
| --- | --- |
| **Buffer 10X** (Provided with the Taq kit) | 2 µl (1X) |
| **Primer fwd.** | 0.2 µM |
| **Primer rev.** | 0.2 µM |
| **dNTP** | 200 µM each |
| **MgCL2** | 1.5 mM (contained in the buffer 10X) |
| **BSA** | 0.8 µl |
| **DMSO** | 4% v/v |
| **gDNA template** | 2 µl |
| **Ultrapure H_2_O** | 13.1 |
| **Qiagen Taq** | 0.5 unit |
| **Total volume of one reaction** | **20 µl** |

**First round cycling conditions**

| **Step** | **Temperature** | **Time** | **Repeats** |
| --- | --- | --- | --- |
| Initial denaturation | 95 ^o^C | 3 mins | 1X |
| Denaturation | 95 ^o^C | 45 s | 40X |
| Annealing | 55 ^o^C | 30 s |  |
| Extension | 72 ^o^C | 45s |  |
| Final extension | 72 ^o^C | 10 mins | 1X |

**Second round reagents**

| **Component** | **Volume** |
| --- | --- |
| **DNA** (amplicons from first round) | 5 µl |
| **Nextera XT index primer 1 (N7xx)** | 2.5 µl |
| **Nextera XT index primer 2 (S5xx)** | 2.5 µl |
| **Kappa HiFi HotStart ReadyMix** | 12.5 µl |
| **Ultrapure H_2_O** | 2.5 µl |
| **Total volume of one reaction** | **25 µl** |

**Second round cycling conditions**

| **Step** | **Cycling conditions** | **Time** | **Repeats** |
| --- | --- | --- | --- |
| Initial denaturation | 95 ^o^C | 3 mins | 1X |
| Denaturation | 95 ^o^C | 30 s | 8X |
| Annealing | 55 ^o^C | 30 s |  |
| Extension | 72 ^o^C | 30s |  |
| Final extension | 72 ^o^C | 5 mins | 1X |

**ITS amplification**

**First round reagents**

| **Component** | **Final concentration** |
| --- | --- |
| **Buffer 10X** (Provided with the Taq kit) | 2 µl (1X) |
| **Primer fwd.** | 0.2 µM |
| **Primer rev.** | 0.2 µM |
| **dNTP** | 200 µM each |
| **MgCL2** | 1.5 mM (contained in the buffer 10X) |
| **BSA** | 0.8 µl |
| **DMSO** | 4% v/v |
| **gDNA template** | 2 µl |
| **Ultrapure H_2_O** | 13.1 |
| **Qiagen Taq** | 0.5 unit |
| **Total volume of one reaction** | **20 µl** |

**First round cycling conditions**

| **Step** | **Temperature** | **Time** | **Repeats** |
| --- | --- | --- | --- |
| Initial denaturation | 95 ^o^C | 3 mins | 1X |
| Denaturation | 95 ^o^C | 45 s | 40X |
| Annealing | 45 ^o^C | 30 s |  |
| Extension | 72 ^o^C | 45s |  |
| Final extension | 72 ^o^C | 10 mins | 1X |

**Second round reagents**

| **Component** | **Volume** |
| --- | --- |
| **DNA** (amplicons from first round) | 5 µl |
| **Nextera XT index primer 1 (N7xx)** | 2.5 µl |
| **Nextera XT index primer 2 (S5xx)** | 2.5 µl |
| **Kappa HiFi HotStart ReadyMix** | 12.5 µl |
| **Ultrapure H_2_O** | 2.5 µl |
| **Total volume of one reaction** | **25 µl** |

**Second round cycling conditions**

| **Step** | **Cycling conditions** | **Time** | **Repeats** |
| --- | --- | --- | --- |
| Initial denaturation | 95 ^o^C | 3 mins | 1X |
| Denaturation | 95 ^o^C | 30 s | 8X |
| Annealing | 55 ^o^C | 30 s |  |
| Extension | 72 ^o^C | 30s |  |
| Final extension | 72 ^o^C | 5 mins | 1X |

**Section III**

**Setting used in JMP**

When testing for significance of bioaugmentation and plantation, we first assessed the normality of the dataset using the “distribution” analysis. Data were transformed if needed. The ANOVA model was built using the “Fit model” option and looked like follows: “Bioaugmentation”, “Plantation”, “Bioaugmentation x Plantation”. We also considered the block from which the samples came and marked it as a random factor. Once we established the significance of the factors, we compared the means using Tukey’s HSD. For the effect of bioaugmentation on plant biomass, we used Student’s T test to compare the two means for each plant species (with or without bioaugmentation).

**R code for the vegan 2.5 package functions used for the analysis**

**Permanova**

**library** (vegan)

**#Import OTU table**

ITS_perm = read.delim("ITS_FINAL4000_permanova.txt", header=TRUE, row.names=1)

**#Import Metadata file**

ITS_Meta = read.delim("ITS_Meta_permanova.txt", header=TRUE, row.names=1)

**# Hellinger transformation to deal with double zeros**

ITS_perm_hel = decostand(ITS_perm, "hel")

**# Generate Bray-Curtis dissimilarity matrix**

ITS_bray_perm = vegdist(ITS_perm_hel, "bray")

**#Actual permanova**

**#Testing factors and their interaction**

- adonis(ITS_bray_perm~Inoculation*Plant_Presence*Plant_Species, data = ITS_Meta, strata = ITS_Meta$Block, permutations = 999)

**#Testing factors without interaction**

- adonis(ITS_bray_perm~Inoculation+Plant_Presence+Plant_Species, data = ITS_Meta, strata = ITS_Meta$Block, permutations = 999)

**#Generating PCoA data**

Bac.pcoa<- cmdscale(bacteria_bray_perm, k=(nrow(bacteria_perm_hel)-1), eig=TRUE)

Bac.points <-Bac.pcoa$points[,1:2]

Bac.eig<-Bac.pcoa$eig

Bac.pcoa$eig[1:2]/sum(Bac.pcoa$eig)*100

Then use the ordiplot() command to draw and customize.

**RDA Analysis**

**#Data**

ITS_OTU<-read.delim("ITS_FINAL4000_permanova.txt", row.names=1, header=T, dec=".")

ITS_metadata2F<-read.delim("ITS_Metadata_RDA.txt",row.names=1, header=T, dec=".")

ITS_metadata2F<-ITS_metadata2F[,1:3]

str(ITS_metadata2F)

**#Transform the data to get rid of the double-zero problem**

ITS_OTU_hel2F<-decostand(ITS_OTU, "hel")

**#RDA**

ITS_rda_all2F<-rda(ITS_OTU_hel2F ~ ., data=ITS_metadata2F )

summary(ITS_rda_all2F)

**#Calculate R2**

(R2adjITS2F<-RsquareAdj(ITS_rda_all2F)$r.squared)

**#Verify collinearity of factors (if two factors are collinear, must eliminate one of them)**

vif.cca(ITS_rda_all2F)

**#Test significance of Whole model**

anova.cca(ITS_rda_all2F, step=999)

**#Test which RDA axes are significant**

anova.cca(ITS_rda_all2F, by="axis", step=999)

**#Test which factor is significant**

anova.cca(ITS_rda_all2F, by="term", step=999)

**#Plot if needed**

plot(ITS_rda_all2F, scaling=2, main="Fungi RDA - scaling 2", type="none", cex.axis=1.5, cex.lab=1.5, cex.sub=1.5, xlab=c("RDA1"), ylab=c("RDA2"), xlim=c(-0.6,0.6), ylim=c(-1.1,0.4))

**##### Betadispertion**

data("ITSbetadispPlanted1")

##Transform hellinger

ITSbetadispPlanted = decostand(ITSbetadispPlanted1, "hel")

## Bray-Curtis distances between samples

dis <- vegdist(ITSbetadispPlanted)

## Designate groups

groups <- factor(c(rep(1,45), rep(2,45)), labels = c("Planted","Planted_Inoculated"))

groups <- factor(c(rep(1,8), rep(2,8)), labels = c("Not_Planted","Not_Planted_Inoculated"))

## Calculate multivariate dispersions

mod <- betadisper(dis, groups)

mod

plot(mod)

## Perform test

anova(mod)

## Permutation test for F

permutest(mod, pairwise = TRUE)

## Tukey's Honest Significant Differences

(mod.HSD <- TukeyHSD(mod))

plot(mod.HSD)

## Plot the groups and distances to centroids on the

## first two PCoA axes

plot(mod)

## Draw a boxplot of the distances to centroid for each group

boxplot(mod)
